# Incubation of methamphetamine craving in punishment-resistant individuals is associated with activation of specific gene networks in the rat dorsal striatum

**DOI:** 10.1038/s41380-024-02455-2

**Published:** 2024-02-14

**Authors:** Atul P. Daiwile, Michael T. McCoy, Bruce Ladenheim, Jayanthi Subramaniam, Jean Lud Cadet

**Affiliations:** Molecular Neuropsychiatry Research Branch, NIH/NIDA Intramural Research Program, Baltimore, MD 21224 USA

**Keywords:** Neuroscience, Molecular biology, Psychology

## Abstract

Methamphetamine use disorder (MUD) is characterized by loss of control over compulsive drug use. Here, we used a self-administration (SA) model to investigate transcriptional changes associated with the development of early and late compulsivity during contingent footshocks. Punishment initially separated methamphetamine taking rats into always shock-resistant (ASR) rats that continued active lever pressing and shock-sensitive (SS) rats that reduced their lever pressing. At the end of the punishment phase, rats underwent 15 days of forced abstinence at the end of which they were re-introduced to the SA paradigm followed by SA plus contingent shocks. Interestingly, 36 percent of the initial SS rats developed delayed shock-resistance (DSR). Of translational relevance, ASR rats showed more incubation of methamphetamine craving than DSR and always sensitive (AS) rats. RNA sequencing revealed increased striatal Rab37 and Dipk2b mRNA levels that correlated with incubation of methamphetamine craving. Interestingly, Bdnf mRNA levels showed HDAC2-dependent decreased expression in the AS rats. The present SA paradigm should help to elucidate the molecular substrates of early and late addiction-like behaviors.

## Introduction

Methamphetamine (METH) is a highly addictive psychostimulant with a very high prevalence of misuse throughout the world [1]. Many, but not all, METH users meet diagnostic criteria for METH use disorder (MUD) [2–4]. These criteria include excessive drug taking during binges and compulsive METH use despite adverse consequences [2]. Compulsive use is thought to be a fundamental feature of addictive diatheses [5–7]. However, there is no FDA-approved medication to treat individuals with MUD. The development of pharmacological therapeutics against MUD might depend on the elucidation of METH-induced molecular and biochemical changes that occur in various cortical and subcortical brain regions that subserve reward, decision-making, and habit forming [8–16]. The fact that only a subset of METH users meets criteria for MUD [2–4], suggests the presence of differences in the molecular substrates in the reward circuitries that control the development of MUD in various individuals. A brain region of special interest is the dorsal striatum, which serves as a key regulator of habit forming [17] and participates in the development and progression of compulsive drug taking in models of substance use disorders (SUDs) [18–23]. The dorsal striatum might also serve as a node in neuronal pathways involved in the tenacity of misuse of licit and illicit substances despite nefarious consequences associated with their abuse [24]. The role for the dorsal striatum in METH-induced behavioral consequences is supported by observations of epigenetic and transcriptional changes in the dorsal striatum of rats that had self-administered the drug compulsively over several weeks [10, 25–27].

In order to clarify the role of striatal molecular mechanisms in compulsive METH taking, we have conducted studies in rats that meet the DSM5 criterion of compulsive drug taking in the presence of adverse consequences represented by contingent footshocks during METH self-administration (SA) experiments [25, 28–32]. We have reported that rats which continue to take METH compulsively in the presence of footshocks showed greater incubation of METH craving than animals that had suppressed their METH intake [13, 31, 33]. We also found that compulsive SA behaviors are also correlated with differences in the balance between orbitofrontal and prelimbic striatal circuits of rats [30], findings that are clinically relevant in view of the relevance of these circuits in SUDs [34–36]. Moreover, there was differential expression of genes and proteins in the dorsal striatum of compulsive and non-compulsive rats [13, 37], potentially implicating that structure in the behavioral differences observed in these two METH SA phenotypes.

The present study was undertaken to investigate the time course of punishment sensitivity over several weeks and to identify genes and molecular pathways associated with these behaviors by using a genome-wide gene expression platform. Several groups of investigators have used genome-wide approaches to identify genes of interest in animal models of cocaine [38, 39], heroin [16], and morphine [40] use, as well as in the case of psychiatric diseases [41]. Herein, we provide convincing evidence for the existence of rats that develop persistent punishment-resistant METH SA over several weeks. We also report that a subpopulation of rats that had initially suppressed their drug intake in the presence of shocks developed delayed shock resistance (DSR) after 2 weeks of forced abstinence. Global analysis of gene expression by RNA sequencing revealed the presence of many differentially expressed genes (DEGs) in the dorsal striatum of compulsive (addicted) versus non-compulsive (non-addicted) rats. Finally, chromatin immunoprecipitation studies identified HDAC2 as an upstream epigenetic regulator of the expression of some trophic factors including *Bdnf* that had shown decreased expression in the non-compulsive rats.

## Materials and methods

### Animals and intravenous surgery

Male Long Evans rats weighing 350–400 g were purchased from Charles River, USA. They were group-housed with free access to food and water. We performed intravenous surgery as described in our previous publications [28, 42, 43]. More details are provided in *supplementary text section 1*. All animal procedures were approved by the National Institute of Drug Abuse Animal Care and Use Committee (Protocol No. 21-MNPB-10) and conducted according to the Guide for the Care and Use of Laboratory Animals (ISBN 0-309-05377-3).

### METH self-administration

METH dose (0.1 mg/kg/infusion), METH SA training procedures (three 3-h sessions/day separated by a 30-min off interval between each session) for 20 days under a fixed-ratio-1 (FR-1) schedule, footshock phase, and METH seeking tests on withdrawal day 1 (WD1) and 15 (WD15) were performed according to published protocols [13, 28, 44]. As described previously 50% of the reinforced active lever presses resulted in the simultaneous delivery of a 0.5-s footshock through the grid floor [28]. The *always* shock-resistant (ASR) and shock-sensitive (SS) rats were separated as per our previous published studies [28, 33]. The detailed METH SA training procedures are described in *supplementary text section 2*.

**Fig. 1:**
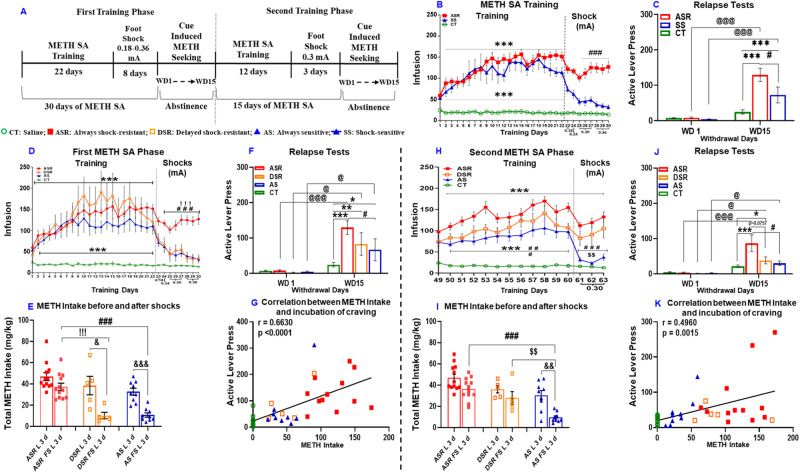
Extended METH self-administration (SA) and contingent footshocks result in compulsive METH taking in a subpopulation of rats. **A** Experimental timeline showing long access METH SA sessions and contingent footshock sessions. Footshocks were administered randomly during 50% of pressing the active lever for METH. **B** During the first phase of the experiment, footshocks reduced lever pressing in shock-sensitive (SS, n = 14) but not in always shock-resistant (ASR, *n* = 12) or compulsive rats. Always shock-resistant (ASR) and shock-sensitive (SS) rats were separated as per our previous published study in which we classified animals as shock-sensitive if they reduced their intake by 60% [33]. **C** Resistant/compulsive rats showed greater incubation of METH craving than SS rats at withdrawal day 15 (WD15) during forced abstinence. **D** SS rats were separated post-facto during the second phase of METH SA training because a subset of the SS rats, now labeled ‘Delayed shock resistance (DSR)’, developed shock-resistance during the second phase of the behavioral experiments. We named the remaining SS rats ‘Always sensitive (AS)’ (see **H**). **E** Footshocks caused marked reduction in METH intake in the sensitive rats during the first phase of footshocks. The figure shows METH intake during the last 3 days of training without shocks (L 3 d) and the last 3 days of the first footshock phase (FS L 3 d). **F** ASR rats showed greater incubation of METH craving in comparison to AS rats but not DSR rats during tests of relapse during the first forced abstinence period. **G** Regression analysis shows a positive correlation between METH intake during first sets of footshock days and active lever responding during the relapse test on WD15 of the first phase of the behavioral experiments. **H** Footshocks reduced lever pressing for METH in the AS rats but not in the ASR and DSR rats. **I** Footshocks reduced METH intake in the AS but not in the ASR and DSR rats. **J** Persistently compulsive SR (ASR) rats continued to show incubation of METH craving in comparison to AS rats during the second set of relapse tests. **K** Regression analysis showed a positive correlation between METH intake during footshock days and active lever responding on the second WD15 relapse test. CT, saline; ASR, always shock-resistant; SS, shock-sensitive, DSR, delayed shock-resistant; and AS, always sensitive rats. Key to statistics: *p < 0.05, **p < 0.01, ***p < 0.001, comparisons between METH groups (ASR, DSR, AS) and controls; #*p* < 0.05, ##*p* < 0.01, ###*p* < 0.001, comparisons between ASR and AS or SS; ! *p* < 0.05, !!*p* < 0.01, !!!*p* < 0.001, comparison between ASR and DSR; $*p* < 0.05, $$*p* < 0.01, $$$*p* < 0.001, comparison between DSR and AS; &*p* < 0.05, &&*p* < 0.01, &&&*p* < 0.001, comparison between before vs after footshocks; @*p* < 0.05, @@*p* < 0.01, @@@*p* < 0.001, comparison between WD1 and WD15.

At the end of the SA plus contingent sessions, rats underwent METH seeking tests at withdrawal days 1 and 15 (see Fig. 1A). Following these tests, rats were placed back inside their respective SA chambers for a second METH SA training phase that lasted for 12 days (Fig. 1A). During the last 3 days of the second METH training phase, rats were again exposed to contingent footshocks at an intensity of 0.30 mA. Subsequently, rats were removed from the SA boxes and housed individually in the animal vivarium with no access to METH. They then underwent a second set of cue-induced METH seeking on withdrawal days 1 (WD1) and 15 (WD15) in their respective SA chambers.

### Tissue collection

Rats were euthanized 24 h after the drug seeking test on WD15 by rapid decapitation with a guillotine. Using specific neuroanatomical coordinates obtained a rat atlas, dorsal striata (dSTR, A/P + 2 to −2 mm bregma, M/L ± 2 to 5 mm, D/V −3 to −6 mm) were dissected out and immediately snap-frozen on dry ice and stored at −80 °C.

### RNA Sequencing

Total RNA was isolated from the dSTR using Qiagen RNeasy Mini kit (Qiagen, USA) and quantified with nanodrop. RNA integrity (RIN) was checked using the Agilent bioanalyzer 2100. RNA samples with RIN 8 or above were shipped on dry ice to Azenta, Genewiz (USA) for RNA sequencing (More details are provided in s*upplementary text section 3*). RNA sequencing data have been deposited at the NCBI under the accession # GSE220896.

### Quantitative polymerase chain reaction (qPCR)

We used qPCR to validate the changes in expression of several genes of interest identified as differentially expressed in the RNA-Seq analysis. Advantage RT-for-PCR kit (Clontech) was used to reverse transcribed 500 ng of total RNA using oligo dT primers. Using iQ SYBR Green Supermix (Bio-Rad, USA), qPCR was performed with Roche LightCycler 480 II system. The relative mRNA expression was normalized to beta-2-microglobulin (B2M). The qPCR primer sequences used in the study are listed in supplementary Table 1.

### Chromatin immunoprecipitation (ChIP) and PCR

Chromatin immunoprecipitation using an antibody specific for HDAC2 was carried out as per our previously published protocol [45]. More details are also provided in *supplementary text section 4*. Real-time qPCR was also performed with the Roche LightCycler 480 II system using promotor specific ChIP-PCR primers listed in Supplementary Table 1. The primers were procured from the Synthesis and Sequencing Facility of Johns Hopkins University.

### Statistical Analyses

Behavioral mRNA and ChIP-PCR data were analyzed with the statistical program GraphPad Prism 9 using ANOVA with repeated measures and followed by Fishers protected least significant difference (PLSD) test. The detailed statistical analyses are described in *supplementary text section 5*.

## Results

### A subpopulation of rats exhibits compulsive METH taking despite punishment

Figure 1A illustrates the timeline of our behavioral experiment. We analyzed the behavioral data using repeated measures two-way ANOVA with group (saline vs METH) and training days (22 days) as factors. The effects of groups [F (1, 36) = 67.18, *p* < 0.0001], training days [F (21, 756) = 7.411, *p* < 0.0001] and their interaction [F (21, 756) = 8.030, *p* < 0.0001] were significant (Supplementary Fig. 1A). During the first punishment phase, some rats (*n* = 12) exhibited continuous compulsive METH intake despite increasing shock intensity from 0.18 to 0.36 mA over 8 days; these rats were termed always shock-resistant (ASR) or compulsive drug takers (Fig. 1B). The observations of persistent resistance to punishment are comparable to the results of Giuliano et al. (2018) who had reported that compulsive alcohol seeking emerged in a subpopulation of rats in the presence of footshock punishment and persisted for almost a year [46]. In contrast to the punishment-resistant animals, other rats (n = 14) decreased lever pressing during the punishment phase and were named shock-sensitive (SS, *n* = 14) or non-compulsive (Fig. 1B). Rats were classified as shock-sensitive if they reduced their intake by more than 60% [33].

We used these two different METH phenotypes (ASR and SS) to conduct further statistical analysis using two-way ANOVA for the first 22 days of METH SA. The analysis revealed significant effects of training days [F (21, 504) = 17.67, *p* < 0.0001], but no significant effect of groups (ASR, SS) [F (1, 24) = 0.3708, *p* < 0.5483] nor their interaction [F (21, 504) = 1.283, *p* < 0.1798] (Fig. 1B and Supplementary Fig. 1B). When we analyzed the behavior for ASR and SS during the first footshock phase, we observed significant effects of groups [F (1, 244) = 32.53, *p* < 0.0001], footshock days [F (10, 240) = 28.23, *p* < 0.0001] and their interaction [F (10, 240) = 5.493, *p* < 0.0001] (Fig. 1B and Supplementary Fig. 1C).

### Compulsive METH takers showed increased propensity to relapse

At the end of the first punishment phase, rats underwent METH-seeking tests under extinction conditions at withdrawal days (WD) 1 and WD15. Drug or food-seeking has been shown to increase gradually during periods of abstinence, a behavioral phenomenon termed “incubation of craving” [47–49]. These behaviors are measured by recording number of active lever presses in the presence of cues previously paired with METH infusions. Drug-seeking gradually increases during the period of abstinence and is clinically relevant because similar phenomena have been observed in human METH users [50]. Two-way ANOVA revealed significant effects for WD [F (1, 24) = 44.14, *p* < 0.0001], but only a significant trend for groups (CT, ASR, SS) [F (1, 24) = 3.790, *p* = 0.0634] and their interaction [F (1, 24) = 3.790, *p* = 0.0768]. Compulsive rats also displayed higher active lever pressing than non-compulsive rats on WD15 (Fig. 1C).

### Some non-compulsive (SS) rats display compulsive METH use upon re-exposure to punishment after 15 days of forced abstinence

After the first abstinence period, rats were again allowed to self-administer METH. Both ASR and SS rats significantly increased their METH intake over the period of 12 days of METH SA (Fig. 1H). Rats were then re-exposed to a second contingent shock phase (shock intensity of 0.30 mA). As per above, rats that reduced their METH intake by more than 60% were classified as shock-sensitive [33]. Unexpectedly, we found that about 36% (*n* = 5) of the SS rats did not suppress their METH intake as they did during the first footshock phase (Fig. 1D); we labeled these animals ‘delayed shock-resistant’ (DSR) rats (Fig. 1H). The rest of SS rats continued to suppress their METH intake (Fig. 1H) as they did before (Fig. 1D) and we labeled those rats, ‘Always sensitive’ (AS), to separate them from the DSR and the SS rats. Importantly, the initially resistant rats (ASR) continued to self-administer METH in a compulsive fashion despite the footshocks (Fig. 1H).

The behavioral breakdown during the second punishment phase led us to re-analyze the data using saline and three METH self-administering phenotypes (ASR, DSR, and AS) as variables. We found that, during the first 22 days of SA, there were significant effects for groups (ASR, DSR and AS) [F (3, 34) = 23.97, *p* < 0.0001], training days [F (4.054, 137.8) = 21.64, *p* < 0.0001] and group by training days interaction [F (63, 714) = 4.767, *P* < 0.0001] (Fig. 1D).

Analysis of the first punishment phase documented that ASR rats continued to self-administer METH whereas DSR and AS rats decreased their METH intake in the presence of footshocks. ANOVA revealed significant effects for groups (ASR, DSR and AS) [F (2, 23) = 22.67, *p* < 0.0001], footshock days [F (3.562, 81.92) = 5.205, *p* = 0.0014] and their interaction [F (14, 161) = 2.408, *p* = 0.0044] (Fig. 1D). ASR rats showed significant differences in their METH SA in comparison to DSR and AS rats (Fig. 1D). In addition, total METH intake was significantly higher for ASR rats in comparison to DSR and AS rats during the last three days of footshocks (Fig. 1E).

METH seeking behaviors for ASR, DSR and AS rats were measured during forced abstinence. ANOVA revealed significant effects for withdrawal day [F (1, 34) = 42.79, *p* < 0.0001], groups (CT, ASR, DSR, and AS) [F (3, 34) = 5.532, *p* = 0.0033] and their interaction [F (3, 34) = 6.512, *p* = 0.0013]. ASR, DSR, and AS rats significantly increased their active level responding on WD15 in comparison to WD1 (Fig. 1F). ASR rats maintained significantly higher active lever responding in comparison to AS but not DSR rats (Fig. 1F). Regression analysis between METH intake during footshock phase and active lever responses on WD15 revealed a positive correlation (r = 0.6630, *p* < 0.0001) (Fig. 1G).

ANOVA for the second METH SA phase revealed significant effects for groups (CT, ASR, DSR and AS) [F (3, 34) = 40.58, *p* < 0.0001], SA days [F (11, 374) = 19.05, *p* < 0.0001] and SA days x groups interaction [F (33, 374) = 4.963, *p* < 0.0001] (Fig. 1H). The total METH intake was also significantly higher in ASR rats when compared to AS rats (Supplementary Fig. 1D). There were no significant differences in total METH intake between ASR vs DSR for that phase (Supplementary Fig. 1D).

ANOVA for the second punishment phase identified significant effects for groups [F (2, 23) = 19.30, *p* < 0.0001], punishment days [F (1.882, 43.28) = 22.32, *p* < 0.0001] and their interaction [F (6, 69) = 3.374, *p* = 0.0056]. Both ASR and DSR rats continue to self-administer METH despite footshock punishment and self-administered significantly higher METH infusion during the three days of footshocks in comparison to AS rats (Fig. 1H). ASR and DSR had significantly higher total METH intake than AS rats during three days of footshocks (Fig. 1I).

### Persistently compulsive METH takers continued to show greater propensity to relapse

Relapse tests were conducted for a second time after forced abstinence. ANOVA revealed significant effects of withdrawal day [F (1, 34) = 22.58, *p* < 0.0001], groups [F (3, 34) = 3.183, *p* = 0.0362] and their interaction [F (3, 34) = 3.411, *p* = 0.0283]. Post hoc test showed that ASR, DSR and AS rats significantly increased their active level responding on WD15 compared to WD1 (Fig. 1J). ASR rats had significant higher active lever responding on WD15 in comparison to CT and AS animals but not DSR rats (*p* = 0.0757) (Fig. 1J). There were positive correlations between active lever responses on WD15 of the second set of relapse tests against total METH intake during the second footshock phase (Fig. 1K, r = 0.4960, *p* < 0.0015) and total METH intake for all rats throughout the experiment (Supplementary Fig. 1E,r =  0.5170, *p* < 0.0009).

Based on these data, we reasoned that these behavioral differences might be associated with molecular differences in brain regions that subsume habit forming and/or acquisition of sequential behaviors. We thus performed RNA sequencing to identify potential transcriptional changes in the dorsal striatum that might be specifically associated with the different behaviors observed in the three groups of METH rats.

### RNA sequencing identifies specific differentially expressed genes in the dorsal striatum of always-resistant, delayed-resistant, and always-sensitive rats

**Fig. 2:**
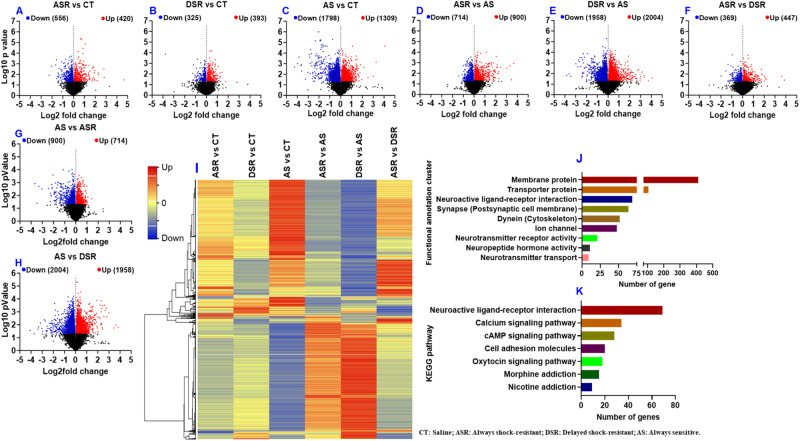
RNA sequencing revealed large-scale changes in gene expression in the dorsal striatum. Analysis of raw sequencing data using Log2 fold changes and log10 *p* values revealed many differentially expressed genes in pairwise comparisons shown as volcano plots: (**A**) ASR vs CT, (**B**) DSR vs CT, (**C**) AS vs CT, (**D**) ASR vs AS, (**E**) DSR vs AS, (**F**) ASR vs DSR, (**G**) AS vs ASR and (**H**) AS vs DSR. **I** Hierarchical clustering of 1194 differentially expressed genes across six pair-wise comparisons. The red color represents over-expressed genes, the blue color represents genes with reduced expression, while the yellow color represents genes with no changes in expression. **J** Functional gene clusters and (**K**) KEGG analysis show pathway distribution of differentially expressed genes according to DAVID.

To identify potential gene networks involved in compulsive and suppressed behaviors in response to punishment, we used RNA sequencing to measure global transcriptional changes in the dorsal striatum (dSTR) of rats euthanized 24 h after the second WD15 relapse test. Results of RNA sequencing are illustrated in Fig. 2. Using DESeq2, we performed comparisons of gene expression between eight different pair-wise comparison (ASR vs CT, DSR vs CT, AS vs CT, ASR vs AS, DSR vs AS, ASR vs DSR, AS vs ASR, and AS vs DSR); the results are shown as volcano plots in Fig. 2. Analysis of raw sequencing data using Log2 fold changes and log10 p values revealed the number of genes with higher or lower expression in ASR vs CT (Fig. 2A), DSR vs CT (Fig. 2B), AS vs CT (Fig. 2C), ASR vs AS (Fig. 2D), DSR vs AS (Fig. 2E), ASR vs DSR (Fig. 2F), AS vs ASR (Fig. 2G), and for AS vs DSR (2H) comparisons.

We also used a more restrictive cut-off of equal or greater than 1.5-fold (*p* = 0.05) and used Qiagen Ingenuity Pathway software to identify gene networks that might have been affected in the different groups of METH rats. The number of differentially impacted genes is provided for ASR vs CT, DSR vs CT, AS vs CT, ASR vs AS, DSR vs AS, and ASR vs DSR in the Venn diagrams (Supplementary Fig. 2A, B) and the changes in the expression of these 1194 genes are illustrated in the hierarchical clustering heat-map shown in Fig. 2I.

**Fig. 3:**
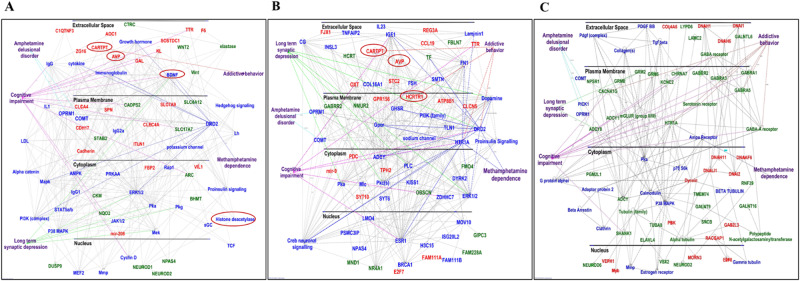
Ingenuity pathway analysis (IPA) identifies several networks of DEGs in compulsive and non-compulsive METH takers in comparison to the control groups. **A** Pathways and genes significantly enriched in the ASR vs CT comparison. **B** Pathways and genes significantly enriched in the DSR vs CT comparison. **C** Pathways and genes significantly enriched in the AS vs CT comparison. The red color indicates upregulated genes, the green color represents downregulated genes, and the blue color represent interacting gene partners.

The Database for Annotation, Visualization and Integrated Discovery (DAVID) was also used to generate functional annotation and clustering for these 1194 differentially expressed genes (DEGs). Functional gene clusters and KEGG pathways are illustrated in Figs. 2J, K. IPA network for the ASR vs CT, DSR vs CT and AS vs CT comparisons were shown in Fig. 3A, B, and C.

### Specific striatal genes are differentially expressed in rats prone to relapse

**Fig. 4:**
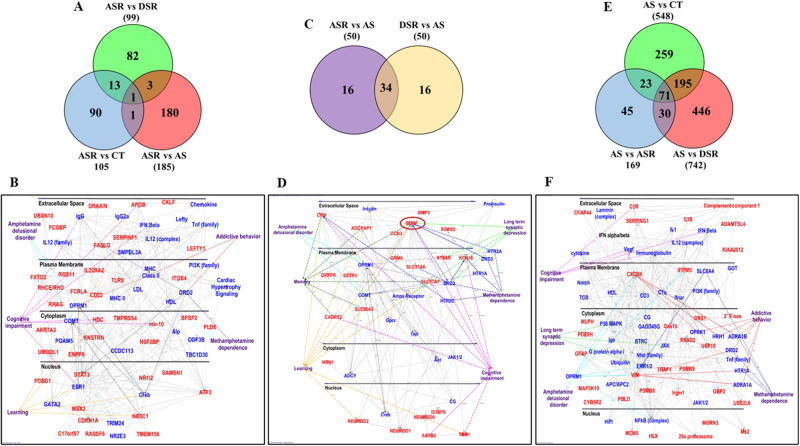
IPA analysis of unique DEGs in the dorsal striatum of ASR, DSR, and AS rats. The Venn diagram in (**A**) illustrates common and unique upregulated gene between 3 pairwise comparisons ASR vs (CT, DSR and AS). **B** Networks significantly enriched among 82 unique genes in the ASR vs DSR comparison. **C** The Venn diagram illustrates the distribution of 50 genes with the highest fold in the ASR compared to DSR or AS rats. **D** IPA analysis shows that 34 shared between ASR vs AS and DSR vs AS comparisons are involved in processes that mediate memory, learning, cognition, and METH dependence. Cognitive impairments are common in patients with MUD. **E** The Venn diagram illustrates the number of genes that are commonly and uniquely located in 3 pairwise comparisons of AS vs (CT, ASR and DSR). **F** IPA shows networks involving 101 genes that are common in AS vs CT, AS vs ASR, and AS vs DSR comparisons (71 genes) and in AS vs ASR and AS vs DSR comparisons (31 genes). These genes are involved in amphetamine delusional disorder, cognitive impairments, and addictive behaviors. The red color indicates upregulated genes, and the blue color represents interacting genes.

One of the important problems associated with the clinical course of MUD is frequent relapses. Relapses have been modeled in animal models using the incubation of drug craving paradigm [43, 47, 48, 51, 52]. In our study, ASR rats showed greater incubation of METH craving that the DSR and AS rats. We used pairwise comparisons to identify genes that were uniquely expressed in ASR rats in comparison to other groups (Fig. 4A). Among these genes are *Mybpc1, Dipk2b, Rab3, and Tnfsf8*. Figure 4B illustrates IPA analysis for genes uniquely found in ASR vs DSR. Supplementary Fig. 3B, C also show IPA analyses for the 180 unique genes in the ASR vs AS comparison. The DEGs uniquely impacted in the ASR groups participate in learning, cognition, addictive behavior, and METH dependence. These results are consistent with behaviors like cognitive impairments reported in patients with diagnosis of MUD [53].

Figure 4C shows 34 genes with higher expression that were common in the ASR and DSR groups in comparison to the AS group. Figure 4D and Supplementary Fig. 4 illustrate the involvement of several of these genes in learning and memory formation, cognitive processes known to be impaired in MUD patients [53].

### Specific striatal genes are differentially expressed in always sensitive (AS) rats

We also sought to identify genes that were impacted in the always sensitive (AS) rats. We used the following comparisons: AS vs CT, AS vs ASR, and AS vs DSR, and identified 71 common that had higher expression in the AS group in comparison to the other group (Fig. 4E). These genes included *Kcnk16*, *Cfap43*, *Cfap44*, *DnaH1*, *DnaH7*, *and DnaI2*, among others. Thirty genes with higher expression in the AS in comparison to ASR and DSR groups are also shown in Fig. 4E). We reasoned that these genes might be involved in inhibiting drug taking behaviors in the presence of adverse consequences. The IPA analysis showed the involvement of some of these genes in learning, cognition, depression, delusional disorder, and addictive behavior (Fig. 4F).

### PCR validation of some genes identified by RNA sequencing analysis

**Fig. 5:**
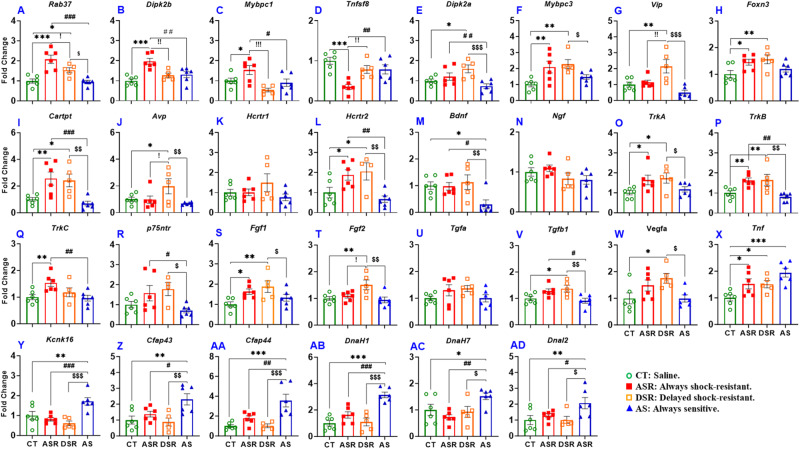
PCR validation of genes identified by RNA sequencing analysis as differentially expressed in various pairwise comparisons between the four groups of rats. Expression of (**A**) *Rab37*, (**B**) *Dipk2b*, (**C**) *Mybpc1*, (**D**) *Tnfsf8*, (**E**) *Dipk2a*, (**F**) *Mybpc3*, (**G**) *Vip*, (**H**) *Foxn3*, (**I**) *Cartpt*, (**J**) *Avp*, (**K**) *Hcrtr1*, (**L**) *Hcrtr2*, (**M**) *Bdnf*, (**N**) *Ngf*, (**O**) *TrkA*, (**P**) *TrkB*, (**Q**) *TrkC*, (**R**) *p75ntr*, (**S**) *Fgf1*, (**T**) *Fgf2*, (**U**) *Tgfa*, (**V**) *Tgfb1*, (**W**) *Vegfa*, (**X**) *Tnf*, (**Y**) *Kcnk16*, (**Z**) *Cfap43*, (**AA**) *Cfap44*, (**AB**) *DnaH1*, (**AC**) *DnaH7*, and (**AD**) *DnaI2* in CT, saline; ASR, always shock-resistant; DSR, delayed shock-resistant; and AS, always sensitive rats. Key to statistics: *p < 0.05, **p < 0.01, ***p < 0.001, comparisons between METH groups (ASR, DSR, AS) and controls (CT); #*p* < 0.05, ##*p* < 0.01, ###*p* < 0.001, comparison between ASR and AS; ! *p* < 0.05, !!*p* < 0.01, !!!*p* < 0.001, comparison between ASR and DSR; $*p* < 0.05, $$*p* < 0.01, $$$*p* < 0.001, comparison between DSR and AS.

We opted to use quantitative PCR to validate the expression of some of the genes of interest identified in the RNA sequencing data (Fig. 5). ASR rats displayed significant higher levels of *Rab37* [F (3, 19) = 14.68, *p* < 0.0001] (Fig. 5A), *Dipk2b* [F (3, 19) = 10.45, *p* = 0.0003] (Fig. 5B), and *Mybpc1* [F (3, 19) = 5.733, *p* = 0.0057] (Fig. 5C) than CT, DSR, and AS. In contrast, *Tnfsf8* expression was decreased [F (3, 19) = 9.230, *p* = 0.0006] (Fig. 5D) in ASR in comparison to CT, DSR, and AS rats. Another member of the *Dipk2b* family, *Dipk2a* showed increased [F (3, 19) = 5.610, *p* = 0.0063] (Fig. 5E) expression in DSR compared to CT and AS. In addition, mRNA levels for *Mybpc3* [F (3, 19) = 5.346, *p* = 0.0077] (Fig. 5F), but not of Mybpc2 (data not shown), were significantly increased in ASR and DSR rats. Because a previous paper had reported that *Mybpc1* was expressed in VIP-expressing interneurons [54], we measured *Vip* mRNA levels and found significant increases [F (3, 19) = 8.871, *p* = 0.0007] only in DSR rats (Fig. 5G).

Regression analysis between active lever presses in the second WD15 relapse test and PCR-validated genes revealed positive correlations for *Dipk2b* (r = 0.4514, *p* = 0.0306) and *Rab37* (r = 0.5614, *p* = 0.0053) and a negative correlation for *Tnfsf8* (r = −0.4368, *p* = 0.0371) (Supplementary Fig. 5A, B, and C, respectively).

We also used PCR to validate *Foxn3*, *Cartpt*, *Avp*, and *Hcrtr* whose mRNA levels were increased in both compulsive ASR and DSR rats in comparison CT and AS animals (Fig. 3, A, B). These are shown in Fig. 5H–L.

Striatal *Bdnf* mRNA levels showed decreased [F (3, 19) = 4.397, *p* = 0.0165] mRNA expression in AS rats in comparison to the other groups, consistent with the RNA-Seq data (Fig. 5M). Although they were not identified in the RNA sequencing analysis, we opted to measure the expression of some other trophic factors, because members of these trophic factors have been implicated in various SUDs [37, 42, 55, 56]. Changes in their expression might also be related to relapse [57]. The results for these including for Fgf1 and Fgf2 are shown in Fig. 5N–X (see below for statistical analyses). Interestingly, AS rats also exhibited significant increases in *Kcnk16*, *Cfap43*, *Cfap44*, *DnaH1*, *DnaH7*, *and DnaI2* mRNA levels in comparison to the other groups (Fig. 5Y–AD), suggesting that their increased expression might be related to suppression of METH taking behaviors in the presence of punishment and/or decreased propensity to relapse.

### Trophic factor-related genes are differentially expressed in compulsive METH users

As mentioned above, there were significant decreases in *Bdnf* (Fig. 5M) expression in the AS METH groups. In order to provide a more panoramic view of potential changes in these families of trophic factors in the METH groups, we used quantitative PCR to measure the expression of some members of the FGF family of genes that have been reported to be impacted in other models of addiction [42, 58]. *Fgf1* mRNA levels were increased [F (3, 19) = 4.429, *p* = 0.0160] in both ASR and DSR phenotypes (Fig. 5S). *Fgf2* was increased significantly [F (3, 19) = 5.016, *p* = 0.0100] in DSR (Fig. 5T). Elevated levels of *Tgfb1* [F (3, 19) = 4.936, *p* = 0.0106] were seen in DSR and ASR in comparison to AS (Fig. 5V). Moreover, DSR rats showed significantly higher [F (3, 19) = 3.896, *p* = 0.0252] *Vegfa* mRNA levels in comparison to AS and control rats (Fig. 5W). *Tnf* mRNA levels were significantly increased [F (3, 19) = 6.547, *p* = 0.0032] in ASR, DSR, and AS in comparison to CT rats (Fig. 5X). In contrast, *Tgfa* (Fig. 5U)*, Egf, Igf1, Tgfb2, Tgfb3*, and *Fgf9* mRNAs were not impacted by METH SA and shocks (data not shown).

### The expression of trophic factor genes is regulated by HDAC2 recruitment at their promoters

**Fig. 6:**
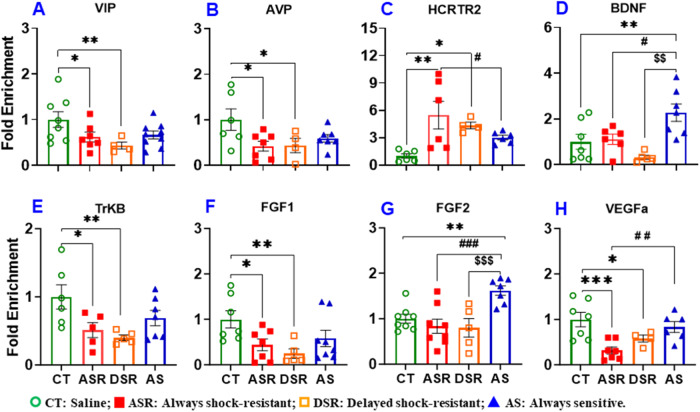
Chromatin immunoprecipitation (ChIP) using a specific HDAC2 antibody identifies several HDAC2-regulated genes in the ASR, DSR, and AS rats. Fold enrichment of: (**A**) VIP, (**B**) AVP, (**C**), HCRTR2, (**D**) BDNF, (**E**) TrkB, (**F**) FGF1, (**G**) FGF2, (**H)** VEGFa. CT, saline; ASR, always shock-resistant; DSR, delayed shock-resistant; and AS, always sensitive rats. Keys to statistics are as described in Fig. 5.

IPA analysis had revealed a potential involvement of histone deacetylases in the regulation of some genes relevant to compulsive drug taking and/or abstinence (see Fig. 3). We had also shown previously that HDAC2 participated in the regulation of METH-induced changes in gene expression in mice [59, 60]. We thus decided to further test the role of HDAC2 in the regulation of some genes of interest by performing chromatin immunoprecipitation (ChIP) followed by qPCR. There were significant decreases [F (3, 24) = 3.040, *p* = 0.0484] in HDAC2 binding at the VIP promoter regions in ASR and DSR (Fig. 6A); these results are partially consistent with the mRNA data that showed increased VIP expression in DSR rats. There were also significant decreases [F (3, 20) = 3.167, *p* = 0.0469] in HDAC2 binding at the *AVP* promoter sequence in the ASR and DSR groups (Fig. 6B) in a manner consistent with the mRNA data (see Fig. 5J). HDAC2 binding at HCRTR2 was also increased [F (3, 17) = 5.026, *p* = 0.0113] in ASR and DSR (Fig. 6C).

Interestingly and importantly, we found significantly increased [F (3, 21) = 6.889, *p* = 0.0021] HDAC2 enrichment at the BDNF promoter sequence in AS animals (Fig. 6D); these observations are consistent with the decreased *Bdnf* mRNA levels in the same rats (Fig. 5M*)*. There was also decreased [F (3, 19) = 40142, *p* = 0.0204] HDAC2 enrichment at the TrkB promoter sequence for ASR and DSR (Fig. 5E), in a manner consistent with the increased mRNA levels in those groups. Other genes for trophic factors showed some interesting changes at their promoters, with FGF1 showing decreased [F (3, 23) = 3.484, *p* = 0.0322] HDAC2 binding for ASR and DSR rats (Fig. 6F) consistent with FGF1 mRNA levels (Fig. 5S), FGF2 showing significantly increased [F (3, 24) = 7.403, p = 0.0011] HDAC2 binding in AS rats (Fig. 6G), and VEGFa showing decreased [F (3, 20) = 6.691, *p* = 0.0026] HDAC2 recruitment in the ASR and DSR (Fig. 6H) groups. The ChIP-PCR data are therefore consistent with the mRNA data (Fig. 5W).

## Discussion

The primary aim of the present study was to assess the longitudinal course of compulsivity and/or abstinence in METH SA rats in response to footshock punishment and to measure the rats’ propensity to relapse during forced abstinence. A secondary aim was to measure global gene expression in the dorsal striatum of the METH SA rats in order to identify specific networks of genes that might be associated with the different behavioral phenotypes. The study confirmed the existence of rats that displayed compulsive METH taking throughout the behavioral experiment despite punishment. These differential responses to METH SA and punishment are consistent with the accumulated evidence that rats show individual differences in their responses to psychostimulants like cocaine and the amphetamines [61–65]. The individual responses to drug self-administration become more prominent in the presence of contingent footshocks [62, 66]. In addition, we found, for the first time, that thirty-six (36%) percent of rats that were initially punishment-sensitive became compulsive METH takers after a second round of METH SA. As previously reported [31, 33], persistently compulsive rats showed significantly higher incubation of METH craving than non-compulsive rats, suggesting that different molecular mechanisms were in play in the brains of these rats. Our RNA sequencing data which identified several genes whose mRNA levels were impacted differentially in the ASR, DSR, and AS rats support this notion, with *Dipk2b* and *Rab37* mRNA levels showing significant positive correlation with incubation of METH craving.

### Development of early and late compulsive behaviors

Rats that showed resistance to footshocks during the first phase of the behavioral experiment remained punishment-resistant even after a period of 2 weeks of forced abstinence. Interestingly, some punishment-sensitive rats became resistant to the footshocks during the second phase of the experiment, supporting the notion that some individuals may be more sensitive to the addictive effects of amphetamine-type substances [67, 68]. The development of delayed resistance to shocks is like the resurgence of behaviors that had been suppressed by punishment or other means [69–74]. Our results are also consistent with the data of Kashani et al. (1987) who had reported that several of their patients had developed SUDs earlier than other patients [75]. Moreover, our behavioral data are compatible with the documentation of two multi-dimensional subtypes of patients who suffer from SUDs [76] including alcohol [77], cocaine [78], and METH [79] use disorders. Altogether, these findings support clinical observations that subpopulations of patients who misuse psychostimulants do exhibit variable clinical courses which might be secondary to different molecular mechanisms. This reasoning suggests the possibility of different approaches to mitigate relapses [72, 73, 80].

### Persistent compulsive METH-taking behavior and proneness to relapse

Rats that showed persistent resistance to punishment throughout the experiment also exhibited greater incubation of METH craving as previously reported [31, 37, 59]. Unexpectedly, rats that developed delayed compulsive METH-taking behaviors during the second set of footshocks failed to show incubation of METH craving as seen in the persistently compulsive rats (see Fig. 1J). These differences in behaviors are supported by the RNA sequencing data that identified genes whose mRNA levels were altered only in the persistently compulsive rats (see Fig. 5A–D). These DEGs include *Dipk2b*, *Rab37*, and *Mybpc1* that showed increased mRNA levels, changes that are correlated to the magnitude of lever pressing for METH during the second set of relapse tests (Supplementary Fig. 5A, B, and D). Dipk2b has been reported to alter neuronal cellular secretory pathways that are involved in autism [81, 82] while RAB37 is known to participate in membrane trafficking and in the regulation of TIMP1 exocytosis [83]. Mybpc1 is thought to contribute to changes in synaptic plasticity [54], which is thought to be an important mediator of the impact of rewarding substances on the brain [7–9, 11]. More experiments, in which the expression of these genes is manipulated, are needed to identify the specific roles that they might play in the development of compulsive METH taking behaviors and incubation of METH craving.

### Delayed METH taking and molecular mechanisms

We identified significant increases in *Dipk2a*, *Avp*, *Fgf2*, *Tgfb1* and *Vegfa* mRNA levels in the dorsal striatum of rats that show delayed resistance to footshocks after METH SA behaviors were suppressed by punishment. The changes in *Avp* mRNA levels are of interest because AVP is known to regulate stress-related behaviors [84, 85] and has been implicated in the development of substance use disorders [86]. The present observations are consistent with those of other studies that had documented increased *Avp* mRNA expression in the nucleus accumbens after exposure to METH [45, 87] and in the amygdala after heroin SA and footshocks [88].

There is some documentation of the participation of trophic factors in the manifestation of drug-taking behaviors. For example, the role for *Fgf2* in SUDs has been well documented [58]. Interestingly, oxycodone self-administering rats showed higher *Fgf2* levels in the dorsal striatum after a month of withdrawal [42]. Of related interest, endogenous FGF2 expression is necessary for the development of sensitization to amphetamine [89]. In addition, the expression of another trophic factor, *Vegfa*, identified in our present study was also increased in the nucleus accumbens of rats after 4 weeks of cocaine administration [90]. Moreover, plasma TGF-beta1 levels were reported to be increased in patients with alcohol use disorders [91]. Altogether, these observations support the idea of the involvement of trophic factors in several models of SUDs.

### Non-compulsive behavior and potential therapeutic approaches

Identifying genes and molecular mechanisms involved in METH addiction is necessary because there is presently no FDA-approved medication for MUD. In the present study, we observed increases in the mRNA levels of potassium channel *Kcnk16*, cilia and flagella associated protein 43 (*Cfap43)* [92], and dynein *(DnaH1, DnaH7, and DnaI2)* [93–96] in the subpopulation of METH SA rats that always reduced their METH intake in the presence of punishment. The increased expression of *Kcnk16* is consistent with the results of our previous experiments in which we also identified increased mRNA expression of potassium channels in shock-sensitive rats [28]. These changes are consistent with findings that potassium channel activators can reduce the intake of rewarding substances (reviewed in 95). Although the potential roles of dynein have not been investigated extensively in models of SUDs, it was recently reported that rats which were chronically injected with heroin showed decreased cortical dynein protein expression [96]. It remains to be determined to what extent manipulations of these genes might influence METH self-administration in the presence of adverse consequences.

### HDAC2 recruitment regulated the expression of genes involved in compulsive behavior

Epigenetic mechanisms that include posttranslational modifications of histone residues participate in the regulation of the expression of plasticity genes that might be responsible for SUD development [26, 97–100]. Histone deacetylases, including HDAC2, that remove acetyl groups from histone residues participate in the regulation of genes that are involved in memory formation and synaptic plasticity [101]. Because HDACs participate in the behavioral manifestations observed in SUD models in animals [26, 102, 103] and in the regulation of the expression of METH-induced immediate early genes in the nucleus accumbens [59], we had reasoned that HDAC2 might regulate the expression of some of the genes identified in the RNA sequencing analysis.

To test the idea, we ran ChIP-PCR to analyze HDAC2 recruitment at some of the mRNAs that we had validated by qPCR. We found that the expression of several trophic factor-related genes including *Bdnf, TrkB, Fgf1, Fgf2* and *Vegfa* showed evidence of regulation by HDAC2 binding at their promoters. Specifically, we found significant increases in HDAC2 binding at the BDNF promoter (see Fig. 6D) and marked decreases in *Bdnf* mRNA levels in the dorsal striatum of AS rats that remained sensitive to the effects of punishment throughout the experiment (see Fig. 5M). The present observations are consistent with those of Guan et al. (2009) who had reported that *Bdnf* expression was regulated by HDAC2, with loss of HDAC2 causing increased *Bdnf* expression in the mouse brain [104]. Moreover, blocking endogenous BDNF expression in nucleus accumbens reduces cocaine SA and relapse [105]. Partial knockout of BDNF also attenuated cocaine seeking behavior in rats [106]. Altogether, these observations support targeting BDNF expression as a potential treatment strategy against MUD.

In addition, we found decreased HDAC2 recruitment at the TrkB promoter which was associated in increased *TrkB* mRNA expression in both compulsive (ASR and DSR) groups. Similar observations were made for *Fgf1* that shows increased mRNA expression in both compulsive (ASR and DSR) rats. Taken together, these data support the involvement of HDAC2 which is known to regulate synaptic plasticity as well as learning and memory processes [101, 104, 107] in regulating the expression of genes that appear to be important in the maintenance of compulsive METH taking in the presence of adverse consequences.

## Conclusion

Our study reports, for the first time shows, that some animals that had initially suppressed their METH intake during a first round of footshocks became resistant to the effects of a second round of punishment. Rats that were resistant to footshocks throughout the experiment showed more incubation of METH craving than other rats. RNA sequencing analysis identified increased expression of some genes including *Rab37* and *Dipk2b* in persistent resistant rats, with their expression showing positive correlation to lever pressing during relapse tests. We also identified increased expression of *Kcnk16*, *Cfap43*, *DnaH1*, *DnaH7*, and *DnaI2* in always sensitive animals. ChIP-PCR identified HDAC2 as a regulator of *Bdnf, TrkB*, and *Fgf1* mRNA levels. Thus, our data are of significant interest because they provide a model that is relevant to the clinical transition from recreational drug taking to compulsive drug taking and addiction that occur with different time courses in human METH users. The RNA sequencing data also identified potential targets for pharmacological interventions against MUD.

## Supplementary information

Supplementary file

## Data Availability

The RNA sequencing data have been deposited at the NCBI GEO under the accession # GSE220896. All other data generated in this study, including PCR, CHIP-PCR, Excel spreadsheets, and GraphPad files, are available upon reasonable request to the corresponding author via email.
