# Supplementary material for: Incubation of methamphetamine craving in punishment-resistant individuals is associated with activation of specific gene networks in the rat dorsal striatum
Source: Mol Psychiatry. 2024 Feb 14;29(7):1990–2000. doi: 10.1038/s41380-024-02455-2 (PMC11408252; doi:10.1038/s41380-024-02455-2)
Supplement: Supplementary file 1 — Supplementary file [file 41380_2024_2455_MOESM1_ESM.docx]

**SUPPLEMENTARY FILE**

Incubation of methamphetamine craving in punishment-resistant individuals is associated with activation of specific gene networks in the rat dorsal striatum.

Atul P. Daiwile, Michael T. McCoy, Bruce Ladenheim, Subramaniam Jayanthi, and Jean Lud Cadet*

*Corresponding author. [jcadet@intra.nida.nih.gov](mailto:jcadet@intra.nida.nih.gov)

**SUPPLEMENTARY TEXT:**

1. **Intravenous surgery**

After the rats were anesthetized with ketamine and xylazine (50 and 5 mg/kg, i.p., respectively), one end of a silastic catheter was placed in their right jugular veins while the other end of the catheter was attached to a modified 22-gauge cannula. This was mounted to the backs of the rats. After the surgery, rats were injected with meloxicam (1 mg/kg, s.c.) to relieve pain and were allowed to recuperate for 5 to 7 days before the start of self-administration (SA). During the recovery and training phases, catheters were flushed with sterile saline and gentamicin (0.05 mg/kg) after every 24 hours.

1. **METH Self-administration**

Male rats were trained in SA chambers (30 × 20 × 20 cm) which were located inside sound-attenuated cabinets and equipped with retractable active and inactive levers above (8.5 cm) the grid floor. Rats were housed in SA chambers where they had free access to food and water throughout the experiment. Rats used in the experiment were drug-naïve and not food-trained before the start of SA experiment. Before the start of the behavioral experiments, rats were randomly assigned to either saline (n = 12) or METH (n = 26) groups. Rats were trained to self-administer METH-HCl (0.1 mg/kg/infusion) for three 3-hr sessions/day separated by a 30-minute off interval between each session for 22 days under a fixed-ratio-1 (FR-1) schedule with 20-second timeouts between each infusion. Presses on the active lever, which also was paired with 5-second compound tone-light cue, triggered the infusion pump to deliver METH (0.1 mg/kg/infusion) over 3.5 seconds (0.1 mL/infusion). After each 3-hour session, the active lever was retracted, and the red house light was turned off. Rats were trained to self-administer METH for 5 days a week with weekends off. During the 2 weekend days, rats lived in the SA chambers, but their intravenous SA lines were disengaged from METH infusion pump assembly. Rats that self-administer saline were treated in a similar way.

During the foot-shock punishment phase, rats continued METH SA every day (three 3-h sessions/day separated by 30 min off intervals) under the FR-1 with 20-second timeout (reinforcement schedule that was used during training). For METH trained rats, 50% of the reinforced active lever-presses also resulted in the simultaneous delivery of a 0.5-s footshock through the grid floor. We set the initial footshock at 0.18 mA, then at 0.24 mA for one day at each intensity. We then increased the intensity to 0.30 mA for next three days and then to 0.36 mA for last 3 days (a total of 8 punishment days). The always shock-resistant (ASR) and shock-sensitive (SS) rats were separated as per our previous published study (29). Specifically, animals were classified as shock-sensitive if they reduced their intake by 60%. Control rats did not receive footshocks.

After 22 days of training phase and 8 days of punishment phase, rats were individually housed in the animal vivarium with no access to METH. They were then tested for cue-induced drug seeking on withdrawal days 1 (WD1) and 15 (WD15). Each morning of the test days, rats were brought back to their respective SA chambers. Each drug seeking test consisted of a 3-hour session, during which presses on active lever continued to present tone and light cues previously paired with METH infusions but did not result in a drug infusion. All rats tested on WD1 were also tested on WD15.

1. **RNA Sequencing**

RNA samples were quantified using Qubit 2.0 Fluorometer (Life Technologies, Carlsbad, CA, USA) and RNA integrity was checked using Agilent TapeStation 4200 (Agilent Technologies, Palo Alto, CA, USA) by Genewiz. The RNA sequencing libraries were prepared using the NEBNext Ultra II RNA Library Prep Kit for Illumina using manufacturer’s instructions (New England Biolabs, Ipswich, MA, USA). Briefly, mRNAs were initially enriched with Oligo d(T) beads. Enriched mRNAs were fragmented for 15 minutes at 94°C. First strand and second strand cDNA were subsequently synthesized. cDNA fragments were end repaired and adenylated at 3’ends, and universal adapters were ligated to cDNA fragments, followed by index addition and library enrichment by PCR with limited cycles. The sequencing libraries were validated on the Agilent TapeStation (Agilent Technologies, Palo Alto, CA, USA), and quantified by using Qubit 2.0 Fluorometer (ThermoFisher Scientific, Waltham, MA, USA) as well as by quantitative PCR (KAPA Biosystems, Wilmington, MA, USA). The sequencing libraries were clustered on three flowcell lanes. After clustering, the flowcell was loaded on the Illumina HiSeq instrument (4000 or equivalent) according to manufacturer’s instructions. The samples were sequenced using a 2x150bp Paired End (PE) configuration. Image analysis and base calling were conducted by the Control software. Raw sequence data (.bcl files) generated from the sequencer were converted into fastq files and de-multiplexed using Illumina's bcl2fastq 2.17 software. One mismatch was allowed for index sequence identification. After investigating the quality of the raw data, sequence reads were trimmed to remove possible adapter sequences and nucleotides with poor quality using Trimmomatic v.0.36. The trimmed reads were mapped to the reference genome available on ENSEMBL using the STAR aligner v.2.5.2b. The STAR aligner uses a splice aligner that detects splice junctions and incorporates them to help align the entire read sequences. BAM files were generated as a result of this step. On an average RNA sequencing resulted in 38229310 number of reads per sample and an average yield per sample was 11469 “Magabases” with bases scoring per sample was Q30 and above (on an average 94). Further, RNA sequencing raw data was mapped to the Rnor6.0 reference genome. After mapping per sample, average total reads were 37756176, total mapped reads were 36666331, percentage of total mapped reads were 97%, unique mapped reads were 33859707, and percentage of unique mapped reads were 90%. Unique gene hit counts were calculated by using feature Counts from the Subread package v.1.5.2. Only unique reads that fell within exon regions were counted. After extraction of gene hit counts, it was used for downstream differential expression analysis. DESeq2 was used for evaluation of gene expression between six different comparisons. The Wald test was used to generate p-values and log2 fold changes. Genes with an p-value < 0.05 and log fold change > 1.5 were called as differentially expressed genes for each comparison. Identified genes were further mapped with Qiagen Ingenuity Pathway software and Database for Annotation, Visualization, and Integrated Discovery (DAVID) was used to generate functional annotation and clustering for significantly differentially expressed genes.

1. **Chromatin immunoprecipitation (ChIP)**

Fresh frozen tissues from the dorsal striatum (dSTR) were minced to approximately 1 mm sized pieces and instantly cross-linked with 1% formaldehyde for 15 min at room temperature. After 15 min, glycine (concentration of 0.125 M) was added to stop the cross-linking reaction, tissues were centrifuged at 1000g, and supernatant was then discarded. Tissues were then washed 5 times with cold PBS containing a protease inhibitor cocktail (Roche) and 1 mM PMSF (Sigma). After the washes, tissues were quickly frozen on dry ice. The fixed dSTR tissues were resuspended in SDS lysis buffer containing a protease inhibitor cocktail (Roche) and 1 mM PMSF (Sigma) and homogenized twice for 10 s using branson sonifier 450. Each tissue sample was then transferred to TPX plastic tube (Diagenode) and sonicated (15 cycles, 30″ ON and 30″ OFF) in the Diagenode Bioruptor device. After sonication, DNA fragmentation sheared range of 300–600 bp was checked on 2 % agarose gel. Dynabeads magnetic G beads (Life Technologies) were incubated with 5 μg of ChIP antibodies directed for HDAC2 (Thermofisher, # PA1861). As a control, dSTR tissues were also incubated with 5 μg nonimmune rabbit IgG (Millipore, 12–370) overnight at 4 °C. Chromatin lysate (60 µg) was diluted in ChIP dilution buffer (Millipore) to a final volume of 1.5 mL, and 100 μL of the pre-immunoprecipitated lysate was saved as “input” for later normalization. The chromatin lysate was then mixed with antibody/magnetic beads and immunoprecipitated with a HDAC2 antibody overnight at 4 °C. On the next day, beads were washed once with low salt, then with high salt followed with LiCl, and Tris-EDTA according to the manufacturer’s instructions. 500 μL of NaHCO3/SDS elution buffer was used to elute the DNA-protein complexes from the beads. We dissociated DNA and protein complex at 65°C for 4 h under high-salt conditions, followed by treatment with RNase A for 30 min at 37 °C and proteinase K for 1 h at 55 °C. Phenol/chloroform was used to extracted DNA and precipitated with ethanol, and finally resuspended in 80 μL of 10 mM Tris pH 8.0.

1. **Statistical Analyses**

Behavioral data were analyzed with the statistical program GraphPad Prism 9 using factorial ANOVA with repeated measures. For the first 22 days of the first training phase, the dependent variable was numbers of infusions on training days; the independent variables were between-subject factors (saline and METH), within-subject factor (training days) and their interaction. If the main effects were significant (p < 0.05), Fishers protected least significant difference (PLSD) tests were used to compare reward types on each training day while maintaining an overall type I error rate of 0.05. For the first shock phase, the dependent variable was numbers of infusions on training days; the independent variables were between-subject factors [always shock-resistant (ASR) and shock-sensitive (SS)], within-subject factor (training days) and their interaction; these analyses were followed by Fishers PLSD post-hoc test. Data from the first relapse test were also analyzed using ANOVA: the dependent variable was active lever presses on withdrawal days 1 and 15 and the independent variables were group (saline, ASR and SS), withdrawal day (1 and 15) and their interaction; these analyses were followed by Fishers PLSD post-hoc tests when significant.

During the second contingent shock phase, 36% (n=5) of the SS rats did not suppress their METH intake to the same degree that they did during the first foot-shock phase; we labeled these animals ‘delayed shock-resistant (DSR)’ rats. The rest of SS rats suppressed their METH intake as before and were labeled ‘always sensitive (AS)’. The initially compulsive rats continued to self-administer METH in a compulsive fashion and were labeled ‘always shock-resistant (ASR)’.

Linear regression analyses were performed to identify any potential correlations between the active lever press on WD15 and METH intake or mRNA expression. The slopes of all the regression lines were calculated using one-way ANOVA.

mRNA and ChIP-PCR data were analyzed using one-way ANOVA followed by Fishers PLSD test. For all analyses, the null hypothesis was rejected at P ≤ 0.05.

**Supplementary Table 1. List of primers used for quantitative PCR**

|  | **RT qPCR Primers** | | |
| --- | --- | --- | --- |
| Sr. No. | Gene Name | Forward Primer | Reverse Primer |
| 1 | *Avp* | CCG AGT GTC GAG AGG GTT TTT | CAG AAT CCA CGG ACT CTT GTG T |
| 2 | *Bdnf* | AGT GTA ATC CCA TGG GTT ACA CGA | CAG GAA GTG TCT ATC CTT ATG AAC CG |
| 3 | *Cartpt* | TGG GAA GAA GAG GGA CT | TAA TTT GCA CAT GCT TCC A |
| 4 | *Cfap43* | ACA AGA AAA TGG AGA TGG AGA TGG A | CCT CGT AGT TGG GTT CAT TTA GGT A |
| 5 | *Cfap44* | ATA CTG AGG ACA AAA GCG TTC TCT | TAA AAG CCA TCT CCC AGA ACT TGA |
| 6 | *Dipk2a* | AAA TAG CAC TGG CAA AAC TAA ACC A | ATG GTA TCC CAA TCT CCA AAG TGA A |
| 7 | *Dipk2b* | GAT ATG CTG AAG TAG TTG ATG CTG G | CTG CTT GGC TGG CAG GAA TCT A |
| 8 | *DnaH1* | ACT ACG TCA TTG CTG TAG AGA TCC | AGC AGG ACT TAC TGG AGA ACT TT |
| 9 | *DnaH7* | ATA AGC TAG AGA AGG CCT TCC ATG | TTA CAG ATC ACT TGG ACA AGA GGG |
| 10 | *DnaI2* | ACA GGT GAA GAT GAC ATG GAT GAA | GAA ACT CAA CTT TGC GCT TTT CAG |
| 11 | *Fgf1* | AGA AAG CCA TCT TGT TTC TC | GAA GCA CTG CTT ACA AAT TCA |
| 12 | *Fgf2* | GAT CCC AAG CGG CTC TA | ACA CTC CCT TGA TGG ACA |
| 13 | *Foxn3* | ACA AAG AGA GGA GTC AGA GTA TTG G | TCA CTC CTG GAG AAA CTT GCA GAA |
| 14 | *Hcrtr1* | CAT GCT CAA GAG AGC AG | CAG GGA GGG CCT ATA AT |
| 15 | *Hcrtr2* | CCG AAA GAA TAT GAG TGG G | GCA GGT GAT GGT CAC AA |
| 16 | *Kcnk16* | TGC CAT CTT AAT GTA GTA ACG GGA | GCT ACC TAG TAC GGA CCT GTT AAG |
| 17 | *Mybpc1* | ATC ACC TGG ATG AAA AAC AAA GTG G | CTT ACT GTG CTA CAA CTT TCA CCT C |
| 18 | *Mybpc3* | AAG ACT ATA ACA CAG GGG AAG AAG G | ACA CAA CAG CTT CTT ATC AAA CAC C |
| 19 | *Ngf* | GGT ACA ATC TCC TTC AAC AGG ACT | GAC ACT GTC ACA CAC TGA AAA CTC |
| 20 | *p75ntr* | GGC CTA TAT TGC TTT CAA GA | TAC TGT AGA GGT TGC CAT CA |
| 21 | *Rab37* | AGC ATG TCC TAC ACA AGA CCA TT | GGT CGA ACT GAA CCA GTA GAG ATG |
| 22 | *Tgfa* | GCT GGG TAT CCT GGT AG | CAA TAC TGA GTG TGG GAA T |
| 23 | *Tgfb1* | AAA GAC ATC ACA CAC AGT | CCA GGA ATT GTT GCT ATA TTT |
| 24 | *Tnf* | GGC ATG GAT CTC AAA G | CTC CGT GAT GTC TAA GTA |
| 25 | *Tnfsf8* | GGA ACC CAG AGAAAC AAA ACT GAA A | TCC ACA CCT GAT TCT ATT TGA CCA T |
| 26 | *TrkA* | TGA GCA GGG ACA TCT ACA | TCC ACA CAT CAC TCT CG |
| 27 | *TrkB* | CAG AGA GCA TCA TGT ACA G | CAC CTC GTT GTT TGA TAG |
| 28 | *TrkC* | ATC ATG TAC CGG AAG TTT ACC ACA | AAT GAC CTC TGT GTT AGA GAG CTG |
| 29 | *Vegfa* | TGA ACT TTC TGC TCT CTT GGG T | TTC ACC ACT TCA TGG GCT TTC T |
| 30 | *Vip* | GGC TGT GAA GAA ATA CTT GAA CTC C | ACC CTT CTC ATC ATT TCT CTA GCT C |
|  | **ChIP PCR Primers** | | |
| Sr. No. | Gene Name | Forward Primer | Reverse Primer |
| 1 | AVP | CTG CTC AGA CAA GGT TTC CC | TCA CCT CTG CCT GCT ACT |
| 2 | BDNF | AAC AAG AGG CTG TGA CAC TAT GCT C | CAG TAA GTA AAG GCT AGG GCA GGC |
| 3 | FGF1 | CAC TCA CCA GGT CAG ATG CTG GTT C | GTT TTG GTG CTT ACC ACA GCA GCA G |
| 4 | FGF2 | ATC CCT CCC CAG TTC AGT TCC TTC T | TGC AAC TTT CTC CCT TCC TGC CTT T |
| 5 | HCRTR2 | GAG GTG ACA ACC CTT CTG GA | GAG GGA ATA CCT ACA CCC GAA A |
| 6 | TrKB | CAA CTG CGG TAG CAG GAC AGT | GTC ACT TCG CCA GCA GTA GC |
| 7 | VEGFa | CCT GGG AAA GGA AAT TGC CCT CAC T | CCT TAC CAG GGT CCA TAG CCT GAG A |
| 8 | VIP | TTG ACA CCT TGG GGA AAA GAG GAA T | GTA CTG AGC TGG GTG TGT GAT GCT T |


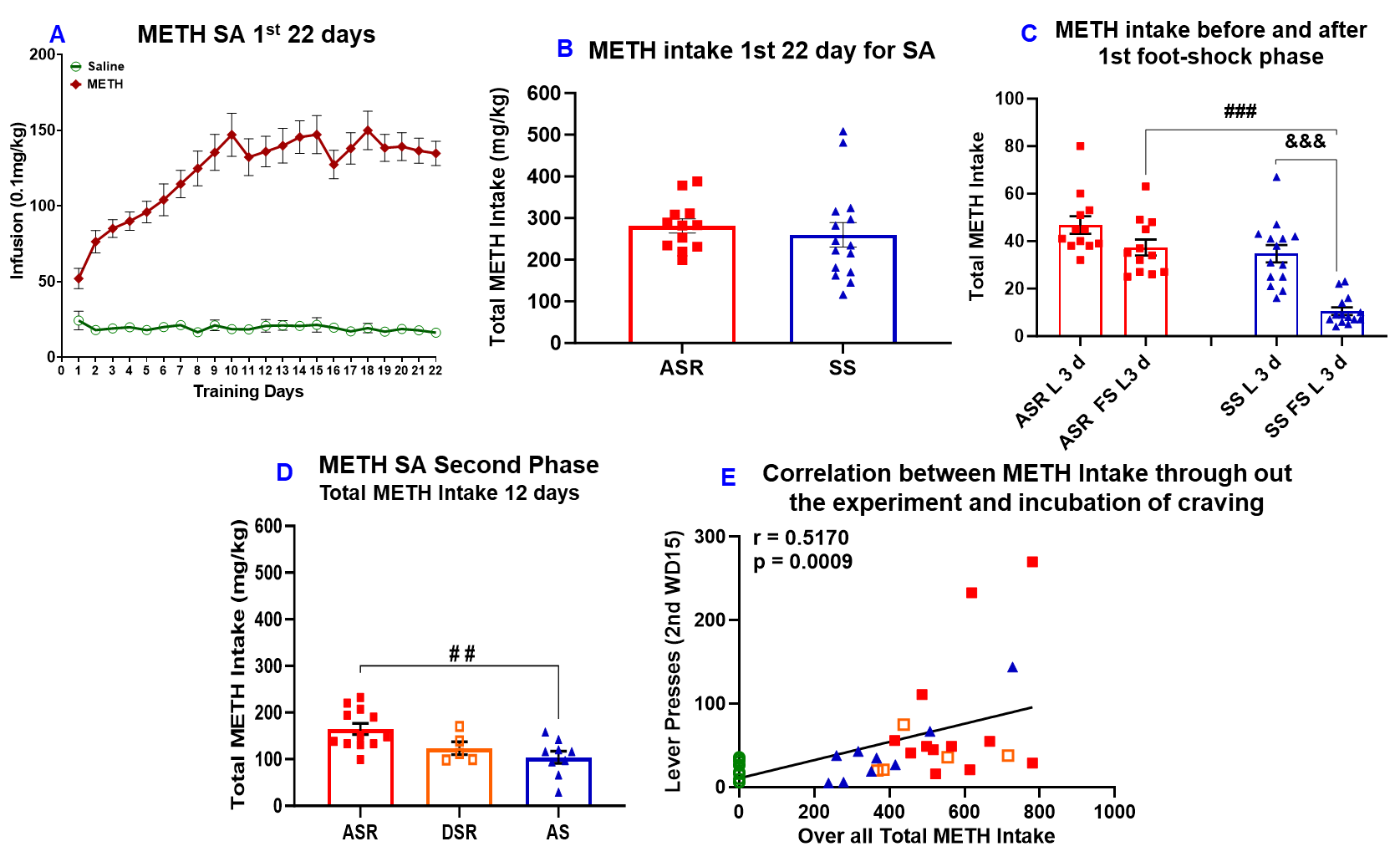


Supplementary Fig. 1. A) Patterns of saline (CT) or METH SA during 22 days of SA training. Total METH intake by ASR and SS rats B) during 22 days of SA and C) during the last 3 days of METH SA training in contrast to METH intake during the last 3 days of the first foot-shock phase. D) Total METH intake by ASR, DSR, and AS rats during the second set of METH SA over 12 days. E) Regression analysis revealed significant a positive correlation between total METH intake during the whole behavioral experiment and active lever responding during the second set of drug METH seeking tests on WD15. Key to statistics is as described in Fig. 1 in the main text.

**
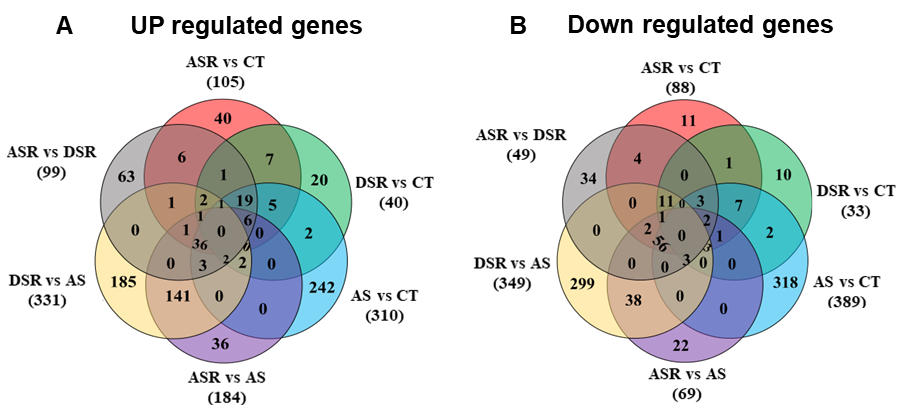
**

**Supplementary Fig. 2.** The Venn diagram in **Figure A** shows genes with higher expression in the groups represented in the numerator whereas the one in **Figure B** illustrates genes with lower expression in the numerator of the 6-pairwise comparisons. For these comparisons, we used more stringent 1.5-fold cut-offs than those used to generate the Volcano plots in Figure 2 in the main text. These genes were used in the hierarchical clustering heat-map shown in Figure 2I.


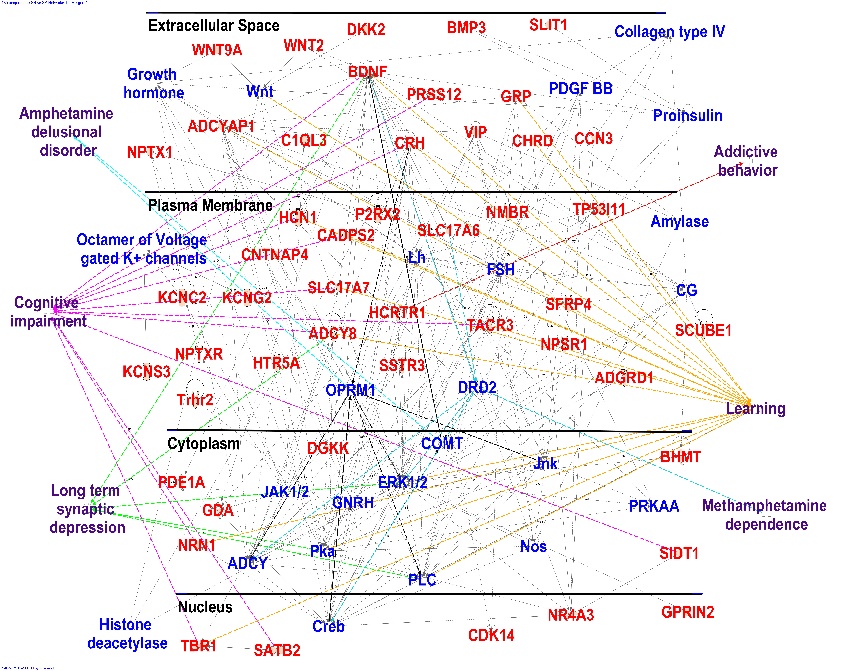

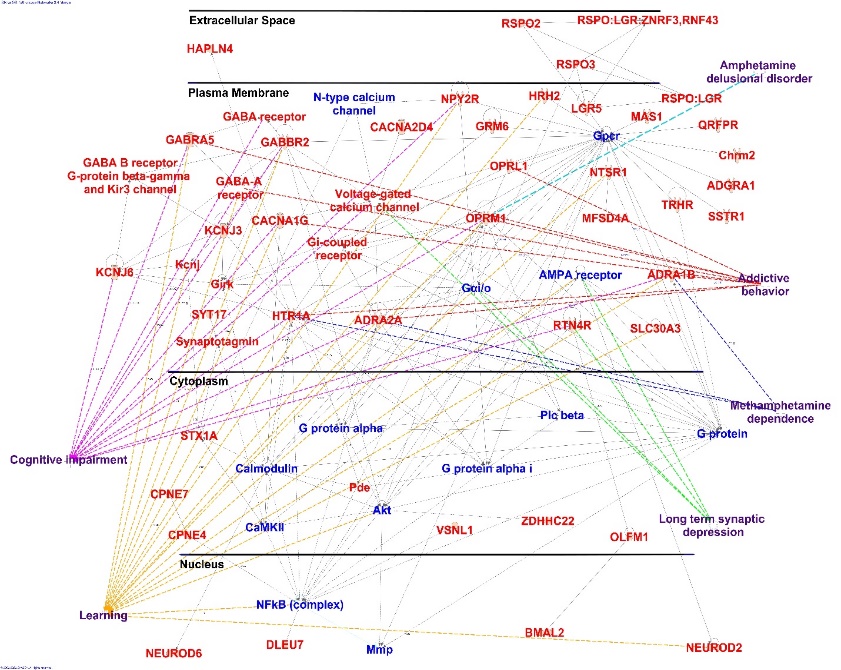


**A**

**B**

**Supplementary Fig. 3. Figures A and B** illustrate 2 IPA analyses using 180 unique genes in the ASR vs AS comparison (see Venn diagram in Fig. 4A in the main text). These genes are involved in processes that mediate learning, cognition, addictive behavior, and METH dependence. The results depicted in these figures are consistent with the idea that several genes can be impacted by rewarding substances, including cocaine, heroin, and morphine, to cause substance use disorders and their neuropsychiatric consequences (see Browne et al., 2023; Campbell et al., 2021; Mews et al., 2023; Mayberry et al., 2022). The red-colored names are genes with higher expression in the dorsal striatum of ASR rats in comparison to the AS rats whereas the blue-colored genes represent their interacting partners according to IPA.

**
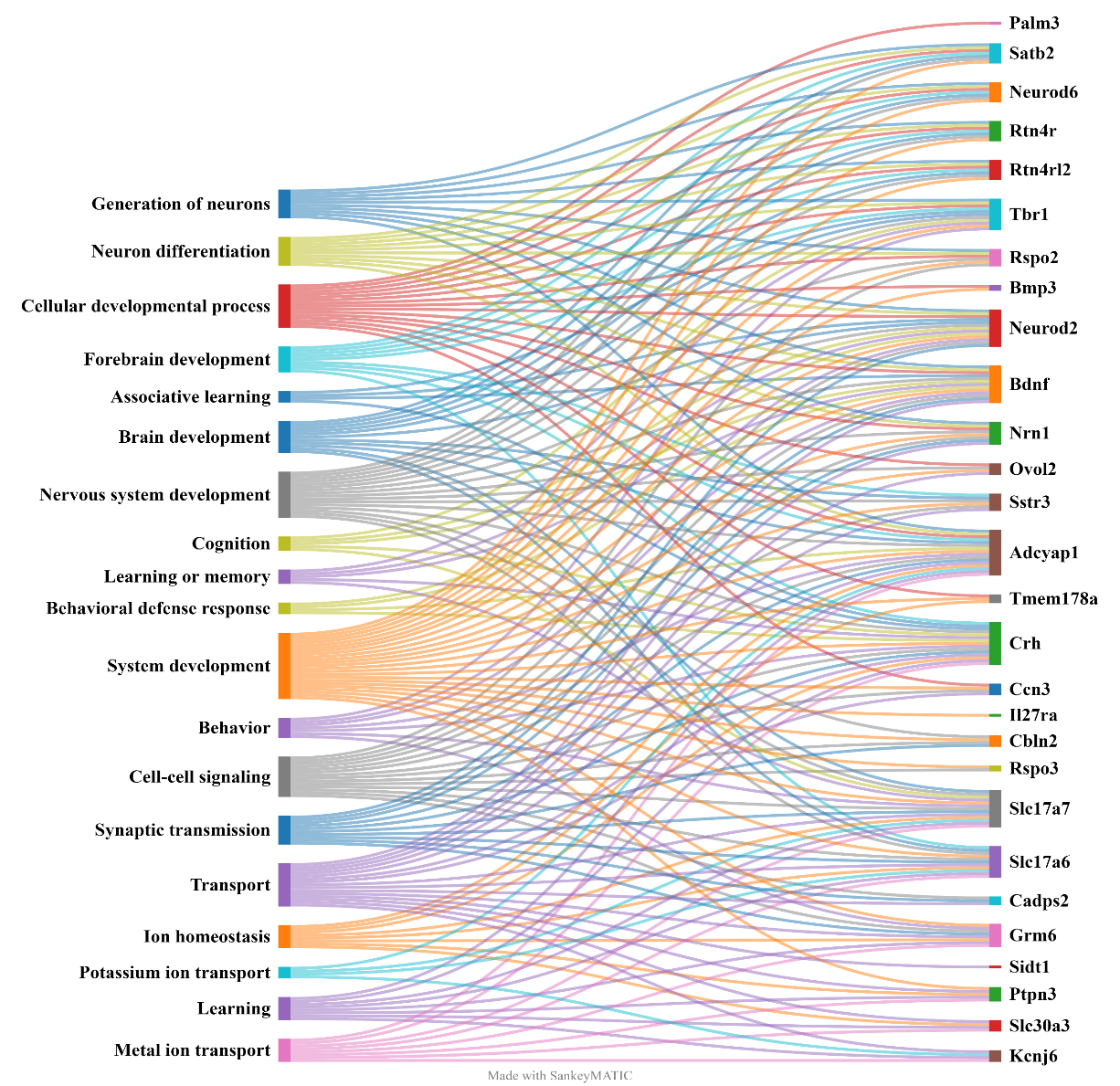
**

**Supplementary Fig. 4.**  The Sankey diagram illustrates the different biological processes for 28 shared genes in higher expression in the ASR and DSR in comparison to the AS phenotype (See Fig. 4C in the main text).


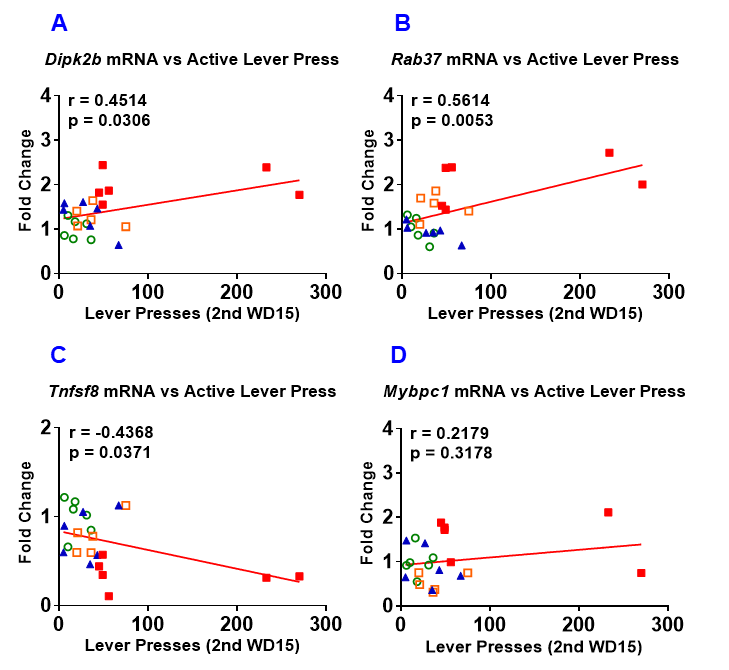


Supplementary Fig. 5. Regression analysis between active lever responding on 2^nd^ WD15 and Fold Change for A) *Dipk2b*, B) *Rab37*, C) *Tnfsf8*, and D) *Mybpc1*.
